# Optimization, Production, Purification and Characterization of HIV-1 GAG-Based Virus-like Particles Functionalized with SARS-CoV-2

**DOI:** 10.3390/vaccines10020250

**Published:** 2022-02-07

**Authors:** Arnau Boix-Besora, Elianet Lorenzo, Jesús Lavado-García, Francesc Gòdia, Laura Cervera

**Affiliations:** Grup d’Enginyeria Cel·lular i Bioprocessos, Escola d’Enginyeria, Universitat Autònoma de Barcelona, Campus de Bellaterra, Cerdanyola del Vallès, 08193 Barcelona, Spain; Elianet.Lorenzo@uab.cat (E.L.); Jesus.Lavado@uab.cat (J.L.-G.); Francesc.Godia@uab.cat (F.G.); Laura.Cervera@uab.cat (L.C.)

**Keywords:** VLP vaccines, HIV-1, SARS-CoV-2, COVID-19, transient transfection, HEK293, design of experiments, bioprocess, downstream process

## Abstract

Virus-like particles (VLPs) constitute a promising approach to recombinant vaccine development. They are robust, safe, versatile and highly immunogenic supra-molecular structures that closely mimic the native conformation of viruses without carrying their genetic material. HIV-1 Gag VLPs share similar characteristics with wild-type severe acute respiratory syndrome coronavirus 2 (SARS-CoV-2) virus, making them a suitable platform for the expression of its spike membrane protein to generate a potential vaccine candidate for COVID-19. This work proposes a methodology for the generation of SARS-CoV-2 VLPs by their co-expression with HIV-1 Gag protein. We achieved VLP functionalization with coronavirus spike protein, optimized its expression using a design of experiments (DoE). We also performed the bioprocess at a bioreactor scale followed by a scalable downstream purification process consisting of two clarifications, an ion exchange and size-exclusion chromatography. The whole production process is conceived to enhance its transferability at current good manufacturing practice (cGMP) industrial scale manufacturing. Moreover, the approach proposed could be expanded to produce additional Gag-based VLPs against different diseases or COVID-19 variants.

## 1. Introduction

COVID-19 is a disease caused by severe acute respiratory syndrome coronavirus 2 (SARS-CoV-2). It emerged in December 2019 in Wuhan and since then has spread around the globe causing a pandemic that had devastating health and economic consequences worldwide [1,2]. An enormous effort made by the scientific community resulted in more than 300 new vaccine candidates in less than a year since the outbreak, some of them being approved for emergency use [3], as well as the development of diagnosis methods for its detection [4] and treatment [5]. As of November 2021, more than 7 billion doses had been administrated [6], with an associated significant reduction of transmission and mortality among vaccinated populations [7]. Vaccination rollout offers a promising avenue for the pandemic and sanitary restrictions to come to an end. However, there are still some questions left to answer, like how long the immune memory lasts, the protective effect that current approved vaccines generate against emerging SARS-CoV-2 variants, or if it is possible to generate fully prophylactic vaccines against this new coronavirus [8].

FDA- and EMA-approved COVID-19 vaccines can be classified into mRNA, adenovirus-based or recombinant. The first group delivers mRNA into dendritic cells (DCs) using a lipid nanoparticle (LNP) as a carrier. The second one delivers DNA into DCs using a non-replicating recombinant adenovirus vector. Both strategies result in DCs producing the genetically encoded SARS-CoV-2 spike (S) surface glycoprotein and presenting it on their own membrane, where it is then recognized by the immune system cells [9]. The third strategy uses a saponin-based nanoparticle to present a recombinantly produced and purified spike glycoprotein, as well [10].

Virus-like particles (VLPs) are a promising, robust, safe, versatile and highly immunogenic approach that can be used to produce novel vaccines for emerging pandemics or diseases, like COVID-19. VLPs are supramolecular structures that closely mimic the native conformation of viruses without carrying genetic material (DNA or RNA), so they are unable to infect, replicate or integrate. They are generated by taking advantage of the intrinsic ability of some viral proteins to self-assemble when expressed in heterologous production platforms. Two VLP-based vaccines have been proven successful and licensed for HPB and HPV [11,12,13]. VLPs can be functionalized to present pathogenic epitopes to generate immunity against diseases like dengue, influenza, etc. HIV-1 Gag VLPs have shown great potential for this purpose, since they are composed by a core of Gag molecules surrounded by a lipid bilayer, a membrane that can be further functionalized [14]. When administrated, due to their tridimensional configuration, VLPs drain and traffic within the immune system, interacting with cells such as DCs, B cells, T cells and macrophages [15,16]. Several proteins present at the VLP membrane interact with DC pattern-recognition receptors leading to a strong adaptative immune response, while multimeric epitopes promote the cross-linking of B cell receptors to induce antibody production [15,16]. Consequently, VLPs had been shown to induce potent humoral and cellular immune responses, and, although the use of adjuvants improves VLP-based vaccines’ immunogenicity, their nature make adjuvant co-administration optional [15]. Therefore, VLP vaccination constitutes a promising approach compared to currently available vaccine technologies: VLPs are safer to manufacture and administrate than inactivated or attenuated vaccines due to their lack of viral genetical material, while they also provide a more potent and effective immune response compared to proteic or subunit vaccines since VLPs present conformationally authentic viral epitopes [17].

Gag VLPs share a similar particle diameter (~145 nm) with wild-type SARS-CoV-2 virus (~80 nm), making them a suitable platform for the expression of its epitopes in order to generate a new vaccine candidate for COVID-19 [14,18]. For this purpose, the SARS-CoV-2 spike (S) protein is a promising lead, since is the major structural protein anchored at the exterior of the membrane of native viruses, carries B cell and T cell epitopes and is the main target for neutralizing antibodies generated from natural infection that protect against viral infection and currently approved COVID-19 vaccines [19,20]. S protein monomers are 180 kDa and assemble to form trimeric units at the surface of native virions, giving them their characteristic crown-shape [21]. They contain a variable receptor-binding domain (RBD), responsible for binding to angiotensin-converting enzyme 2 receptor (ACE-2), facilitating viral entry into target cells [21]. As the disruption of the RBD-ACE2 interaction can block SARS-CoV-2 cell entry [22], most of the reported neutralizing antibodies against SARS-CoV-2 bind to the RBD [23]. 

VLPs can be produced in prokaryotic and eukaryotic heterologous expression systems depending on their nature and use. Mammalian cells constitute an attractive production platform for enveloped or multimeric VLPs due to their capacity to perform complex post-translational modifications. There are several mammalian cell platforms in which VLPs can be produced, such as HeLa, Vero, CAP, CHO or HEK293 [13]. HEK293 can be cultured in suspension in bioreactors using chemically defined media free of animal components and can also be easily transfected. This makes them a good choice to produce Gag-based COVID-19 VLPs in large-scale bioreactors, in order to satisfy the needs for pre-clinical trials, clinical trials, and eventually large-scale production for its manufacture. For this purpose, a robust and scalable downstream process (DSP) needs to be implemented in order to obtain a high-purity vaccine product in its final buffer formulation. Finally, for the initial steps of proof of concept, HEK 293 cells transient transfection methodologies have been well established to provide a robust and fast approach to the generation of VLPs for testing, of special relevance when developing and comparing several candidates.

In the last decades and especially after the COVID-19 outbreak, different published works have focused on the generation of SARS-CoV and SARS-CoV-2 VLPs by the co-expression of the coronavirus S, M and E proteins [24]. This work focuses on the production, purification and characterization of a potential COVID-19 vaccine candidate, based on HIV-1 Gag-based SARS-CoV-2 spike VLPs (from now on S-VLPs), a never-before reported approach to our knowledge. S-VLP production scale-up and its DSP have been achieved by HEK293 transient transfection in a 1 L bioreactor and a purification process consisting of two clarification steps, an ion-exchange affinity step and a size-exclusion polishing and buffer exchange step. The production process and the obtained S-VLPs have been studied and characterized in this work.

## 2. Materials and Methods

### 2.1. Cell Line, Media and Culture Conditions

The serum-free suspension-adapted HEK293 cell line (HEK293SF-3F6) was used, kindly provided by Dr. Amine Kamen from the Biotechnology Research Institute at the National Research Council of Canada (Montreal, Canada) and McGill University. This cell line was derived from a current good manufacturing practice (cGMP) master cell bank available for manufacturing of clinical material.

The medium used for HEK293 cellular growth was the chemically defined and free from animal components HyCell TransFx-H from HyClone (GE Healthcare, Chicago, IL, USA) supplemented with 4 mM GlutaMAX (Gibco, Thermo Fisher Scientific, Waltham, MA, USA) and 0.1% Pluronic F-68 Non-ionic Surfactant (Gibco, Thermo Fisher Scientific, Waltham, MA, USA).

Suspension cell cultures were maintained routinely in exponential growth phase in 125mL or 1L disposable polycarbonate Erlenmeyer flasks with a vent cap (Corning, Tewksbury, MA, USA) in a LT-X Kuhner shaker (LT-X Kuhner, Birsfelden, Switzerland) shaking at 130 rpm, at 37 °C, 5% CO_2_ and 85% RH. Cell counts and viability determinations were performed using the NucleoCounter NC-300 automatic cell counter (Chemometec, Lillerød, Denmark) following the manufacturer’s instructions.

### 2.2. Plasmids and Transfection

#### 2.2.1. Plasmid Expression Vectors

The pGag::eGFP plasmid codes for a codon-optimized Rev-independent HIV-1 Gag protein fused in frame to the enhanced GFP driven by the CMV enhancer and promoter. The plasmid from the NIH AIDS Reagent Program (Cat 11468) (Hermida-Matsumoto and Resh, 2000) was constructed by cloning the Gag sequence from pCMV55M1-10 (Schwartz et al., 1992) into the pEGFP-N1 plasmid (Clontech, Palo Alto, CA, USA).

The pSpike plasmid codes for a mammalian cell codon optimized nucleotide sequence coding for the spike protein of SARS-CoV-2 driven by the CAG enhancer and β-actin promoter. It was produced under HHSN272201400008C and obtained through BEI Resources, NIAID, NIH: Vector pCAGGS Containing the SARS-Related Coronavirus 2, Wuhan-Hu-1 spike Glycoprotein Gene, NR-52310.

pMock plasmid does not have any mammalian promoter or coding DNA sequence (CDS). It was constructed by the ligation of the pGag::eGFP backbone.

#### 2.2.2. Plasmid Amplification and Purification

Plasmids were amplified in *Escherichia coli* DH5α strain grown in LB medium (Conda, Madrid, Spain) supplemented with kanamycin (10 µg/mL, Sigma, St. Louis, MO, USA) or ampicillin (100 µg/mL, Sigma, St. Louis, MO, USA) depending on the *E. coli* antibiotic resistance present on each plasmid. Plasmid purification was carried out using the Endofree Plasmid Mega kit (Qiagen, Hilden, Germany) according to the manufacturer’s instructions.

#### 2.2.3. PEI-Mediated Transient Transfection

Exponentially growing HEK293 cells were passaged in order to have a cell density of 2·10^6^ cells/mL at transfection time. PEIpro (Polyplus-transfection SA, Illkirch-Graffenstaden, France) was used as a transfection reagent. PEI-DNA complexes were formed under sterile conditions, by adding PEI to a plasmid DNA mixture diluted for a total DNA concentration of 1 µg/mL in fresh culture media (10% of the total volume of cell culture to be transfected). The mixture was incubated for 15 min at RT and then added to cell culture. The ratio between plasmids and transfection reagent was optimized using a Box–Behnken design of experiments and described in the next section. 

### 2.3. Box–Behnken Design

A Box–Behnken design was used in order to define the optimal concentration for three independent variables in the cell transfection step: pGag::eGFP, pSpike and PEI. These variables were screened at three levels: a low level, coded as −1; a medium level, coded as 0; and a high level coded as +1, as indicated in Table 1. Box–Behnken experimental results were fitted to a second-order polynomial equation described below by non-linear regression analysis:

Y = β_0_ + ∑ β_i_ X_i_ + ∑ β_ii_ X_i_^2^ + ∑ β_ij_ X_i_ X_j_ (1)

where Y is the response (in this work, the percentage of cells expressing simultaneously Gag::eGFP and spike at 72 hpt); β_0_ is the offset term; β_i_ is the linear coefficient; β_ii_ is the quadratic coefficient; β_ij_ the interaction coefficient, and X_i_ and X_j_ are the independent variables (pGag::eGFP, pSpike and PEI). The equation was used to predict the concentration of the independent variables in order to maximize the desired response. Three-dimensional response surface plots were generated using Design Expert version V8.0.6 software (Stat-Ease Inc., Minneapolis, MN, USA). Statistical analyses of the model were performed using Design Expert. The coefficient values corresponding to the generated response model are shown at Table 1.

Control groups to be transfected with just one plasmid coding for a protein, were co-transfected with pMock plasmid in order to deliver the same gene copies of the protein being expressed in the other conditions. Transfections associated with Box–Behnken optimization studies, validation and bioreactor production were carried out following the later described plasmid and PEI concentrations. Expression was analyzed at 0, 24, 48 and 72 hpt.

### 2.4. Stirred Tank Reactor (STR) Bioprocess

A BioStat B Plus bioreactor (Sartorius Stedim Biotech, Göttingen, Germany) equipped with a 3-blade segment dual impeller with UP-DP configuration [25] was used for HEK293 cell cultivation and production. The agitation was set at 200 rpm; the temperature was set at 37 ºC, and the pH was set at 7.1, controlled with CO_2_ and NaHCO_3_ (7.5% *w/v*). Dissolved oxygen was controlled at 40% of air saturation by supplementing air by sparger at a constant flow of 0.1 L/min and additional pure oxygen when needed. HEK293 growing exponentially in disposable polycarbonate 1 L shake flasks (Corning, Tewksbury, MA, USA) were used to seed the bioreactor at 0.5·10^6^ cells/mL in 1 L of working volume.

### 2.5. Sucrose Cushion Small-Scale Purification

Culture harvests were performed at 72 hpt and centrifuged at 10,000× *g* for 10 min, and the supernatant was stored at −80 °C for further analysis or stored at 4 °C for its purification in less than 24 h. The supernatants containing VLPs were placed on a 30% (*w/v*) sucrose cushion for ultracentrifugation at 31,000 rpm for 2 h at 4 °C. The supernatant was carefully discarded, and pellets were resuspended and placed on a new sucrose cushion for a second ultracentrifugation following the same protocol. The pellets were collected and resuspended in PBS.

### 2.6. Downstream Processing

#### 2.6.1. Cell Harvest and Culture Supernatant Clarification

After 2 h of sedimentation, the culture medium from transfected cells was subjected to primary clarification using Supracap 50 V100 depth filter capsules (Pall Corporation, Port Washington, NY, USA) to remove cellular debris and other contaminants. For secondary clarification, the Supor EAV—Mini Kleenpak 20 filter capsules (Pall Corporation, Port Washington, NY, USA) was used. For both clarifications, a K2Ri pump (Repligen, Waltham, MA, USA) with MasterFlex 96410-13 silicon tubes (Cole-Parmer, Vernon Hills, IL, USA) connected to the filter inlet and outlet; and a pressure sensor (Cole-Parmer, Vernon Hills, IL, USA) connected to the filter inlet. The turbidity of the clarification samples was measured using a portable Eutech TN-100 turbidimeter (Thermo Fisher Scientific, Waltham, MA, USA).

#### 2.6.2. Ion-exchange chromatography (IEX)

A prepacked 0.86 mL Mustang Q XT Acrodisc column (Pall Corporation, Port Washington, NY, USA) was used to capture the S-VLPs from the secondary clarification. Before loading, the column was pre-equilibrated with 5 column volumes (CV) of 5% buffer B (50 mM HEPES, 2M NaCl, pH = 7.2: Buffer B). The sample was directly loaded into the column via the sample pump. After sample application, the column was washed with 5 CV of buffer B at 5%. Elution was achieved by a salt step gradient consisting of 20 CV of 15%, 35%, 45% and 65% of buffer B (300 mM NaCl, 700 mM NaCl, 900 mM NaCl and 1300 mM NaCl). Solutions were filtered using 0.22 μm filters. Chromatographic runs were performed with a flow rate of 1 mL·min^−1^, except for the sample application (10 mL·min^−1^). Fractions of 1 mL were collected and pooled according to the chromatograms.

#### 2.6.3. Size-Exclusion Chromatography

The collected peak containing the desired product from the IEX was loaded into a sepharose 4 Fast Flow (GE Healthcare, Chicago, IL, USA) in-house packed XK 16/40 desalting column of 48 mL. A column performance test with 1% acetone confirmed the correct values of asymmetry 10% and height equivalent to a theoretical plate (HETP). The column was pre-equilibrated with 5 CV of the formulation buffer (20 mM NaH_2_PO_4_, 50 mM NaCl, 2 mM MgCl_2_, 2% sucrose, pH 7.5). Subsequently, the sample was injected onto the column via its sample pump. Elution was achieved with an isocratic elution (0–100%) of 2 CV of the formulation buffer. The column was sanitized with 5 CV of 0.5 M NaOH. The chromatographic run was performed at a 2 mL·min^−^¹ flow rate. Fractions of 1 mL were collected and pooled according to the chromatograms.

### 2.7. Immunocytochemistry Staining for Flow Citometry and Confocal Microscopy

For IF-ICC staining, cells were centrifuged 5 min at 300× *g* and rinsed with staining solution (1.5% (*v/v*) fetal bovine serum (FBS) 1X phosphate-buffered saline (PBS)) before primary antibody incubation for 20 min at 4 °C in the dark. After rinsing twice, cells were incubated with the corresponding secondary antibody for 20 min at 4 °C. After IF-ICC staining, fixation was performed using 2% (*v/v*) formaldehyde 1X PBS for 10 min at RT. Cells were resuspended in staining solution and stored at 4 ºC prior to analysis.

Primary human anti-SARS-CoV-2 spike glycoprotein RBD domain antibody (ab272854, AbCam, Cambridge, UK) was diluted 1:1000. The secondary antibody used for flow citometry analysis was an anti-human IgG (H+L) coupled with Cy™5, produced in donkey (709-175-149, Jackson ImmunoResearch, West Grove, PA, USA), diluted 1:400. The secondary antibody used for confocal microscopy imaging was an anti-human IgG (H+L) coupled with Alexa Fluor 568, produced in goat (#A-21090, Thermo Fisher Scientific, Waltham, MA, USA), diluted 1:400. All IF-ICC antibodies were diluted using staining solution.

#### 2.7.1. Flow Cytometry

The transfected cellular populations of previously IF-ICC stained cells were assessed by flow cytometry using a BD FACS Canto flow cytometer (BD BioSciences, San Jose, CA, USA), at Servei de Cultius Cel·lulars, Producció d’Anticossos i Citometria (Universitat Autònoma de Barcelona, Bellaterra, Catalonia, Spain).

#### 2.7.2. Confocal Microscopy

The imaging of previously IF-ICC-stained cells was performed using Leica TCS SP5 confocal fluorescence microscope (Leica Microsystems, Wetzlar, Germany) at Servei de Microscòpia (Universitat Autònoma de Barcelona, Bellaterra, Catalonia, Spain). Prior to visualization, cells were treated with 0.1% (*v/v*) of Hoechst 33342 (Thermo Fisher Scientific, Waltham, MA, USA) and 0.1% (*v/v*) of CellMask Deep Red (Thermo Fisher Scientific, Waltham, MA, USA) in order to stain cell nuclei and lipid membranes, respectively. Samples were placed in 35 mm glass bottom Petri dishes with 14 mm microwells (MatTek Corporation, Ashland, MA, USA) prior to their visualization under the microscope. 3D images were generated and analyzed using Imaris software (Bitplane, Oxford Instruments, Zurich, Switzerland).

### 2.8. Transmission Electron Microscopy

TEM analyses were performed at Servei de Microscòpia (Universitat Autònoma de Barcelona, Bellaterra, Catalonia, Spain). Samples were visualized in a JEOL 2011 transmission electron microscope (Jeol, Tokio, Japan) operating at an accelerating voltage of 200 kV. Electron micrographs were recorded with the Digital Micrograph software package (Gatan, Pleasanton, CA, USA). Images were recorded by a Gatan US4000 (Gatan, Pleasanton, CA, USA) cooled charge-coupled device (CCD) camera.

#### 2.8.1. Transmission Electron Microscopy: Negative Staining

For negative staining, samples were prepared by means of the air-dried method. Briefly, an aliquot of purified VLPs was absorbed by flotation onto freshly glow discharged 400 mesh carbon film copper grids (22-1MC040-100, MicrotoNano, Haarlem, The Netherlands). After standing for 1 min at RT, excess sample was drained carefully off the grid using Whatman filter paper, Grade 1 (WHA1001325, Merck, Kenilworth, NJ, USA). Samples were then stained with 5µL of uranyl acetate (2%) by incubation for 1 min at RT. The excess uranyl acetate was drained off as previously described.

#### 2.8.2. Transmission Electron Microscopy: Immunogold Labeling

For immunogold labeling, 8 µL of purified VLPs were loaded onto copper grids as previously described. After absorption, two wash cycles were performed. Each wash cycle consisted of adding by flotation 2% (w/v) BSA in PBS and removing the excess sample, followed by the addition of 1X PBS at RT. Then, primary human anti-SARS-CoV-2 spike glycoprotein antibody (ab272854, AbCam, Cambridge, UK) diluted 1:50 was added, and the grids were incubated for 1 h at RT. Following three wash cycles, grids were incubated with 6 nm gold-conjugated anti-human IgG (109-195-088, Jackson ImmunoResearch, West Grove, PA, USA) diluted 1:20 for 1 h at RT. After three wash cycles, grids were stained with uranyl acetate as mentioned before.

### 2.9. Nanoparticle Tracking Analysis

NTA-based Gag::eGFP VLP quantification and characterization was performed using a NanoSight NS300 (Nanosight Ltd., Amesbury, UK) at the soft material services of the Institut de Ciència de Materials de Barcelona (ICMAB-CSIC, Bellaterra, Catalonia, Spain).

### 2.10. Total Protein and dsDNA Quantification

A BCA Protein Assay (#23225, Thermo Fisher Scientific, Waltham, MA, USA) was performed following manufacturer’s instructions using the provided BSA as standard. Colorimetric absorbance at 562 nm was read on a Multilabel Plate Reader Victor3 (Perkin Elmer, Waltham, MA, USA).

A Quant-iT PicoGreen dsDNA Assay Kit (#P11496, Thermo Fisher Scientific, Waltham, MA, USA) was performed following the manufacturer’s instructions using the provided λDNA as standard. Fluorescence (λex= 488 nm, λem= 520 nm) was read on a Multilabel Plate Reader Victor3 (Perkin Elmer, Waltham, MA, USA). The fluorescence value of the reagent blank was subtracted for each sample before calculating the dsDNA concentration using the generated standard curve.

### 2.11. Western Blot and SDS-PAGE

Samples were mixed with 4x Laemili Buffer (#1610747, Bio-Rad, Hercules, CA, USA) and 1.4 M DTT (10708984001, Merck, Kenilworth, NJ, USA) to a final concentration of 1% *v/v*. Each sample was incubated at 96 °C for 20 min and stored at 4 ºC until gelled. Precision Plus Protein WesternC (#1610376, Bio-Rad, Hercules, CA, USA) was used as molecular weight standard. A total of 20 μL of sample per lane was loaded and ran on precast SDS-polyacrylamide (4–20%) gel electrophoresis (#4561093, Bio-Rad, Hercules, CA, USA) at 200V, 400mA for 45 min. Running buffer used was Tris/Glicine/SDS (25 mM Tris, 192 mM glycine, 0.1% SDS, pH 8.3) (#1610772, Bio-Rad, Hercules, CA, USA).

For SDS-PAGE, proteins were stained with Coomassie Brilliant Blue EZBlueTM Gel Staining Reagent (G1041, Sigma Aldrich, St. Louis, MO, USA).

For Western blot, electrophoresis gel was transferred onto a polyvinylidene difluoride membrane for 7 min using the Trans-Blot Turbo Transfer System (#17001918, Bio-Rad, Hercules, CA, USA) following the manufacturer’s instructions. Transferred membranes were then blocked with 5% (w/v) nonfat dry milk in wash buffer (1× PBS 0.1% Tween-20). All the incubations and wash steps between incubations were performed at 40 rpm in a Polymax 1040 rocker shaker (Polymax 1040, Heidolph Instruments, Schwabach, Germany). For anti-HIV-1 Gag WB, blocking was performed overnight at 4 °C and incubated 2 h at RT with primary antibody. For SARS-CoV-2 spike WB, blocking was performed 40 min at RT, and it was incubated overnight at 4 °C with primary antibody. Primary antibodies used were rabbit polyclonal Anti-SARS-CoV-2 spike glycoprotein antibody (ab272504, AbCam, Cambridge, UK) and mouse monoclonal antibody to HIV-1 p24 (A2-851-500, Icosagen, Tartu, Estonia), both diluted 1:1000 in wash buffer. After primary incubation, membranes were incubated using anti-mouse IgG coupled with alkaline phosphatase antibody produced in goat (A3562, Merck, Kenilworth, NJ, USA) or anti-rabbit IgG coupled with alkaline phosphatase antibody produced in goat (A9919, Merck, Kenilworth, NJ, USA), as required, in wash buffer for 1 h at RT. Protein bands were visualized using NBT-BCIP solution (#1706432, Bio-Rad, Hercules, CA, USA) after 2−3 min incubation. Membranes were let to dry and then scanned and analyzed using the software ImageJ2 Fiji (National Institutes of Health, Bethesda, MD, USA).

### 2.12. Dot Blot

Samples were charged into Bio-Dot Apparatus (#1706545, Bio-Rad, Hercules, CA, USA) while a low vacuum was applied. Nitrocellulose membrane (#88018, Thermo Fisher Scientific, Waltham, MA, USA) was placed at the top of humidified filter paper. Once samples were transferred, membrane was incubated with anti-SARS-CoV-2 spike glycoprotein S2 monoclonal antibody (Ab281312, AbCam, Cambridge, UK) and an anti-rabbit secondary antibody (A9919, Merck, Kenilworth, NJ, USA) following the same procedure previously mentioned for Western blot. Once dried, membranes were scanned, and the pixel density for each loaded sample was analyzed using software ImageJ2 Fiji (National Institutes of Health, Bethesda, MD, USA). The standard used for quantification was a recombinant human coronavirus SARS-CoV-2 spike glycoprotein S2 subunit (Ab272106, AbCam, Cambridge, UK).

## 3. Results

### 3.1. SARS-CoV-2 Spike Protein Co-Expression and Localization

Chimeric VLPs were produced in HEK293 cells growing in suspension culture in a chemically defined and animal-component-free media, by transient transfection as a proof of concept. To produce SARS-CoV-2 spike Gag::eGFP VLPs (S-VLPs), cells were co-transfected with plasmids pGag::eGFP and pSpike, using PEI as the transfection reagent. A control cell group was co-transfected with pGag::eGFP and an empty plasmid to generate Gag::eGFP VLPs (from now on, G-VLPs). In order to easily track the HIV-1 Gag polyprotein expression and characterize S- and G-VLPs, Gag was fused in frame with eGFP, as previously reported [26]. S-VLP producer cells showed no significant difference in viable cell density compared with G-VLP producer cells control group (data not shown). Viabilities between 70–80% at 72 hpt were in agreement with values previously observed in PEI-mediated Gag-based VLP productions [26,27]. These results show that the expression of the CoV-2 spike protein does not have a toxic effect on the HEK293 platform used. Otherwise, low viabilities (<70%) could be indicative of toxicity caused by the spike protein’s incomplete maturation through the secretory pathway [28].

**Figure 1:**
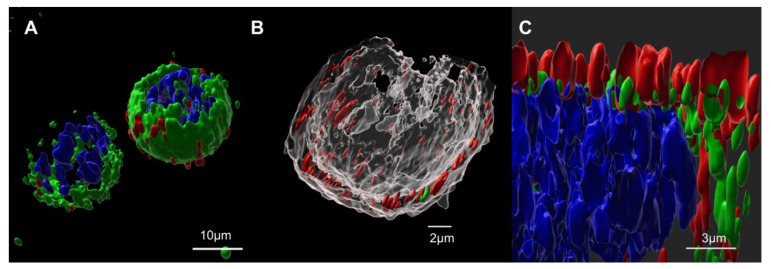
Spike protein cellular localization. Cells were treated with an anti-SARS-CoV-2 S1 spike subunit monoclonal antibody, followed by an Cy5 anti-human secondary incubation. (**A**): Co-localization of spike (red) and Gag (green) can be observed at cells’ external boundary. Cell nuclei were stained and shown as blue. (**B**): By staining the lipid membrane with CellMask, the strong co-localization of S protein (red) and cell membrane (grey) was observed. (**C**): Cross-sectional observation of the plasmatic membrane, where the co-localization of spike (red) and Gag (green) can be observed. Cell nuclei were stained and shown as blue.

Cells were analyzed by confocal microscopy in order to track Gag::eGFP and spike protein localizations at the time of harvest. As can be seen in Figure 1A, the green fluorescence channel shows Gag::eGFP along the cytoplasm to the vicinity of the plasmatic membrane. This corresponds to what was already known about Gag polyprotein maturation, which occurs at the cytoplasm until it reaches the plasmatic membrane surroundings, where budding occurs in order to generate the Gag-based VLPs [29]. To determine spike localization, cells were immunostained using an anti-S primary antibody and a fluorocrome-conjugated secondary antibody. By staining the lipid membrane with CellMask, the strong co-localization of the S protein (red) and cell membrane (grey) was observed (Figure 1B), as well as the co-localization of the S protein (red) and Gag::eGFP (green) in membrane (Figure 1A,C). These results suggest that the expressed S protein could be dragged and incorporated at the surface of the produced S-VLPs, as they bud from the plasmatic membrane [29], where we observe that spike is present.

### 3.2. Characterization of the Produced S-VLPs

**Figure 2:**
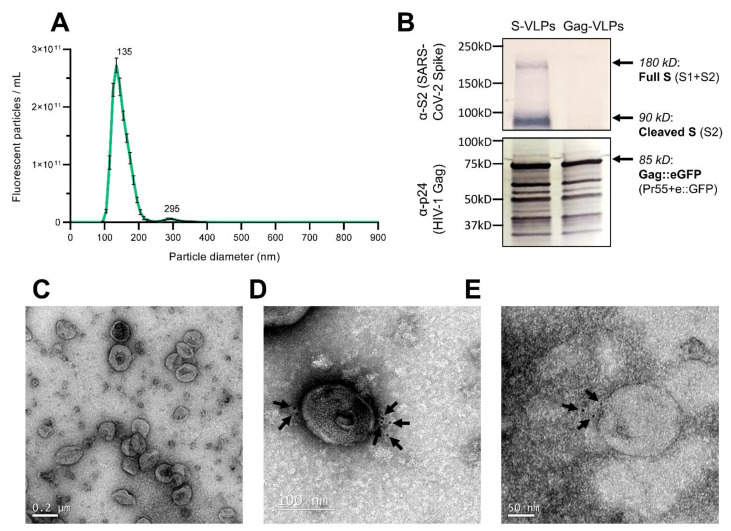
Characterization of the produced S-VLPs. (**A**): Particle-size distribution of the purified S-VLPs, analyzed by NTA. (**B**): Western blot of purified S-VLPs and G-VLPs. Top: Membrane was treated with an anti-SARS-CoV-2 S2 spike subunit polyclonal antibody, followed by a goat anti-rabbit secondary incubation. Bottom: Membrane was treated with an anti-HIV-1 p24 monoclonal antibody, followed by goat anti-mouse secondary incubation. Both S-VLPs and G-VLPs showed HIV-1 Gag bands by Western blot, while only S-VLPs showed intense SARS-CoV-2 protein bands. (**C–E**): Electron microscopy images of the purified VLPs. (**C**): CryoTEM of untreated purified S-VLPs. (**D**): Gold-immunolabeled TEM. (**E**): Gold-immunolabeled CryoTEM. Grids were treated with an anti-S protein monoclonal antibody, followed by incubation with goat anti-human secondary coupled with 6 nm gold particles. Gold-immunolabeled S-VLPs showed S protein localization (arrows) on the surface of the chimeric VLPs.

Small-scale production, followed by sucrose cushion purification, was performed to study if the produced S-VLPs incorporate the S protein. The mode diameter of the purified chimeric S-VLPs was 134.9±1.2 nm, as measured by nanoparticle-tracking analysis (NTA) (Figure 2A). Both purified S-VLPs and G-VLPs showed HIV-1 Gag bands by Western blot, while only S-VLPs showed intense SARS-CoV-2 spike protein bands, as shown in Figure 2B.

The purified VLP concentrates contained spherical enveloped particles with no significant structural differences compared to non-functionalized G-VLPs, as seen under EM by negative staining (Figure 2C). Immunogold labeling using anti-SARS-CoV-2 spike protein primary antibody and 6 nm gold-labeled secondary antibody showed S protein localization on the surface of the chimeric VLPs (Figure 2D,E). These EM and Western blot results confirm the functionalization of the Gag-based produced S-VLPs with SARS-CoV-2 S antigens, which has not been previously described in any published work to the best of our knowledge. Further, we also achieved the generation of a spike presenting enveloped VLP without the need for co-expressing M and E coronavirus proteins, as described in the literature [24].

### 3.3. Transient Transfection Optimization by a Box–Behnken Design of Experiments

Further, the effect of different PEI and plasmid DNA concentrations and their effect on cell transfection were studied using a three-factor, three-level Box–Behnken design of experiments with the aim of finding an optimal condition that maximizes the percentage of cells expressing both Gag::eGFP and spike proteins. The three independent variables at the transfection mix included pGag::eGFP, pSpike and PEI. The experimental design matrix in coded values, response and statistical analysis is shown at Table 1. Experimental data were fitted to a second-order polynomial equation (Equation (1)) using a non-linear regression analysis. The generated equation for the percentage of double-positive transfected cells analyzed by flow cytometry after IF-ICC staining at 72 hpt is shown below (Equation (2)):

Y = 47.3 − 4.763 X_1_ + 4.1 X_2_ − 1.513 X_3_ − 1.175 X_1_·X_2_ + 4.45 X_1_·X_3_ + 0.125 X_2_·X_3_ − 6.1 X_1_^2^ − 3.175 X_2_^2^ + 4.1 X_3_^2^ (2)

where Y is the percentage of double positive transfected cells; X_1_ is the coded value for pGag::eGFP; X_2_ is the coded value for pSpike, and X_3_ the coded value for PEI.

The model fitted the data with a R^2^ of 0.9606, which corroborates its consistency with 96% of the variability in the data. The obtained F-value of 13.54 indicates that the model is also significant. There is only a 0.52% chance that a “Model F-Value” this large could occur due to signal noise. The Fisher’s F-test associated p-value of <0.0052 indicates the model was significant. Values of the terms A, B, AC, A^2^, B^2^ and C^2^ have a “Prob>F” less than 0.05, which also indicates that they are significant.

**Figure 3:**
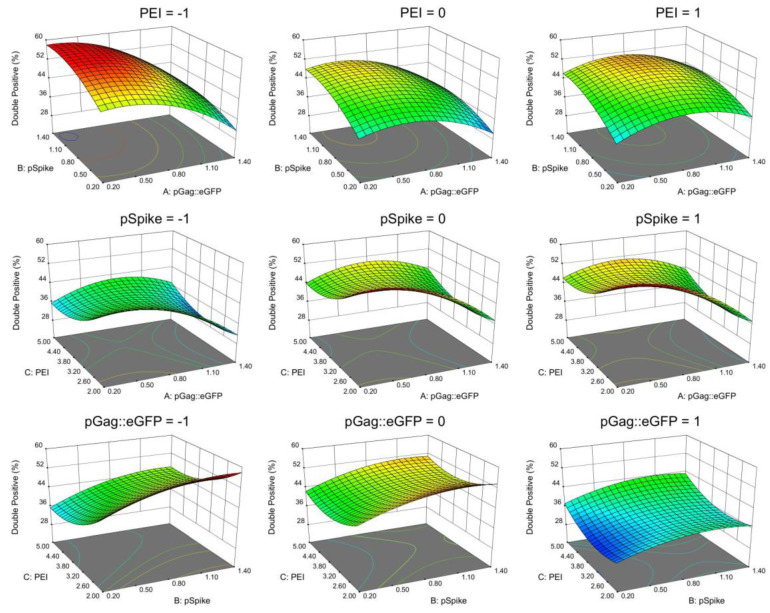
Analysis response surface graphs for the transfection optimization using the Box-Behnken design of experiments. The Y-axis accounts for the analyzed response: the percentage of cells expressing both Gag::eGFP and spike proteins (double-positive population) at 72 h post-transfection. Surface graphs describe the effect of the pGag::eGFP plasmid, pSpike plasmid and PEI concentrations (in µg/mL) in the double-positive population (% of total cells). Each combination of two independent variables is presented at the X- and Z-axis, while the response is shown at the Y-axis. (**Top**): pSpike vs. pGag::eGFP. (**Middle**): PEI vs. pGag::eGFP. (**Bottom**): PEI vs. pSpike. The third independent variable for each pair is modulated at each of the three coded levels (−1, 0, 1), as indicated above each individual surface response graph.

The model was used to plot response surface graphs (Figure 3) and to calculate the optimal factor levels that resulted in the highest double-positive transfected cell population. Response evaluation over the experimental region illustrates that the optimal concentration for pSpike is near the center of the range of concentrations tested, while optimal concentrations for pGag::eGFP and PEI are near their boundaries.

The optimum concentrations found for pGag::eGFP, pSpike and PEI were 0.308 µg/mL, 1.058 µg/mL and 2.045 µg/mL, respectively. With these concentrations, the model predicts 57.5±2.3% of the total cell population would express both Gag::eGFP and spike protein at 72 hpt. To validate the generated model, a verification experiment was performed for the optimal conditions (n=3). A total of 58.9±0.4% of the double-positive population was obtained at 72 hpt, corroborating the predictability of the model and setting the conditions to maximize double-positive cell population.

### 3.4. Production in Stirred-Tank Bioreactor

**Figure 4:**
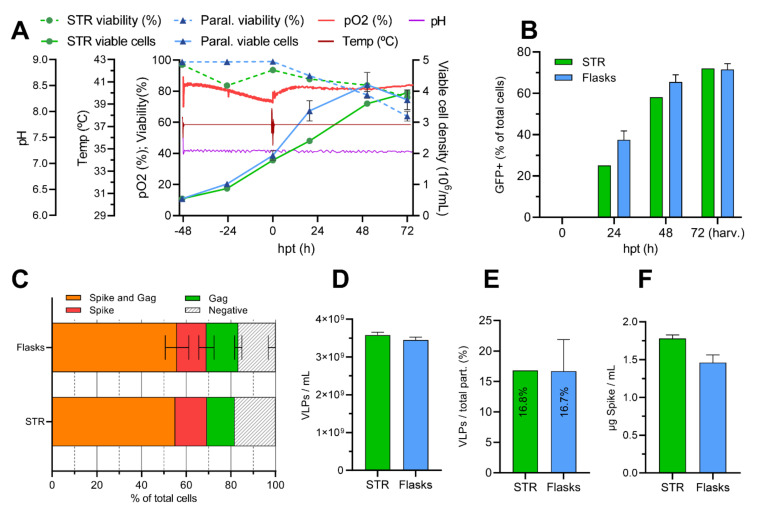
Production of S-VLPs in a stirred 1 L bioreactor and harvest analysis. (**A**): Online profile measurements of different process parameters: Temperature, pH and dissolved oxygen concentration (pO2). Cell density and cell viability values are also shown. The arrow indicates the moment of transfection. (**B**): Transfection kinetics: percentage of Gag::eGFP-expressing cells (single- or double-positive) at different time points after transfection. (**C**): Immunocytochemistry analysis at 72 hpt. STR and shake flasks show a similar percentage of double-transfected populations (55.1% and 55.8%, respectively), together with a ~27% population of single Gag::eGFP-positive cells and a ~13.7% percentage of single S-expressing cells. (**D**): Harvest VLP concentrations, measured by NTA fluorescent particle analysis. (**E**): Harvest purity, which illustrates the percentage of VLPs from the total particles present at the harvest, by NTA fluorescent and non-fluorescent particle analysis. (**F**): Harvest SARS-CoV-2 spike concentrations, analyzed by dot blot.

In order to evaluate production at a bioreactor scale, a culture in a 1L stirred-tank bioreactor (STR) was performed. Cells were inoculated at a concentration of 0.5·10^6^ viable cells/mL (vc/mL). The bioreactor was set to operate at 200 rpm, pH 7.1, 37 ºC and a dissolved oxygen concentration over 40%. The STR culture was transfected with the previously established optimal DNA and PEI concentrations at a cell density of 2·10^6^ vc/mL. After transfection, viable cells continued slowly growing until their harvest at 72 hpt, reaching a final density of 3.95·10^6^ vc/mL, while their viability decreased to 76.1%. As Figure 4A shows, similar behavior was also observed in the parallel runs in 20 mL Erlenmeyer shake flasks (*n* = 3), wherein cells reached slightly higher concentrations of 4.2·10^6^ vc/mL at their peak but with a lower viability of 63.8% at 72 hpt.

The positive green fluorescent population by flow cytometry was evaluated at different time points in order to assess the transfection kinetics. As it can be observed in Figure 4B, fluorescent protein expression during the 48 h after transfection was slightly lower in the reactor in comparison with the Erlenmeyer flasks. At 72 hpt, both reached the same total Gag::eGFP producer population, around 70%. At that time point, culture samples of STR and shake flask were IF-ICC stained and analyzed by flow cytometry in order to assess what percentage of cells was expressing HIV-1 Gag and/or the SARS-CoV-2 spike. Reactor and flasks showed similar percentages of double-transfected cells expressing Gag::eGFP and spike (55.1% and 55.8%, respectively), together with a ~27% population of single Gag::eGFP positive cells and a ~13.7% percentage of single S-expressing cells (Figure 4C). Therefore, no statistically significant differences were observed between the reactor and shake flasks cultures. Viability, growth and transfection analysis allow the conclusion that the production was successfully transferred to a 1 L bioreactor scale, which represents a very promising outcome for a potential scale-up of the process for the production of large amounts of the vaccine candidate for pre-clinical and clinical trials.

Supernatants from the bioreactor and shake flasks had very similar VLP concentrations (≈3.5·10^9^ VLPs/mL) at 72 hpt harvest, with an almost identical level of purity (~16.75% of VLPs over total particles) as evaluated by NTA fluorescent particle analysis (Figure 4D,E). The harvested 1 L work volume of the reactor contained 3.58·10^12^ VLPs with no significant difference in mode particle diameter (data not shown), as evaluated by NTA. The spike concentration of the harvested supernatants was also determined by quantitative dot blot: the reactor showed a concentration of 1.78 µg spike/mL, while flask supernatants had a slightly lower concentration of 1.46 µg spike/mL (Figure 4F).

### 3.5. Downstream Process of the Produced S-VLPs

A downstream purification process (DSP) that could be scalable in order to facilitate its potential use at industrial levels has been considered. For this purpose, a DSP consisting of two clarification steps, a capture ion-exchange step and a polishing size-exclusion final step was tested for the S-VLP purification of the harvested product from the 1L bioreactor.

The two initial clarification steps are necessary since the harvested supernatant presents turbidity caused by cellular debris and contaminating particles that can interfere when loaded into chromatographic columns. After two hours of sedimentation, the harvested media was subjected to primary clarification using a depth filter for the removal of cell debris, intact cells, aggregates, impurities and other contaminating particulate materials from the harvested product. After the primary clarification, the clarified bulk was used as a secondary clarification feed. The filter used in this step is designed for bioburden and particle removal and can favor reductions in the levels of precipitates, as required for proper chromatographic performance. To evaluate the efficacy of the clarification steps, the turbidity was measured during the process. The harvested supernatant had a turbidity of 22.5 NTU after sedimentation. The first clarified bulk showed a turbidity of 4.21 NTU and second clarified 2.13 NTU, which is a desirable value for the good performance of the following purification steps [30].

**Figure 5:**
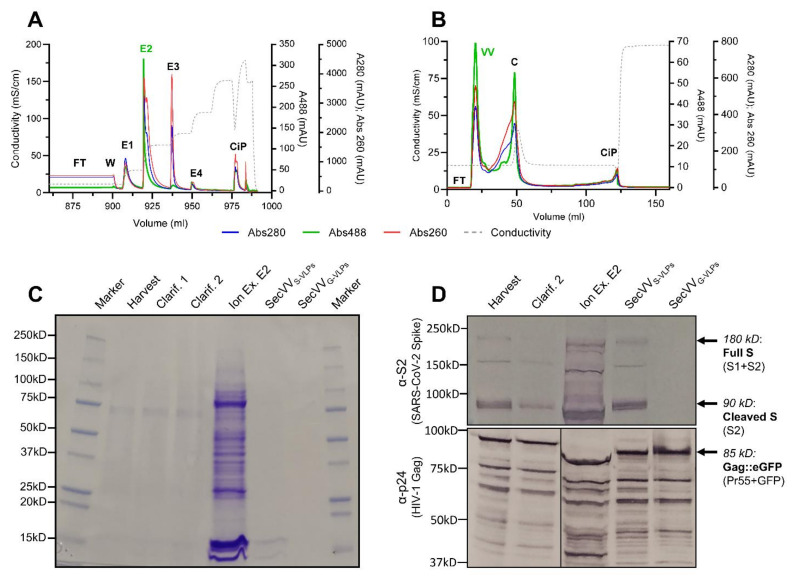
(**A**): Ion-exchange capture step chromatogram: FT (flow through), W (wash), E1-4 (eluted fractions resulting from different conductivity step increases, with E2 being the IEX purified product), CiP (cleaning in place). (**B**): Size-exclusion polishing-step chromatogram: FT (flow through), VV (void volume, which is the final SEC-purified product), C (contaminants and aggregates), CiP (cleaning in place). (**A**,**B**): Blue line: absorbance at 280 nm, indicating protein presence. Red line: absorbance at 260 nm, indicating DNA presence. Green line: absorbance at 488 nm, indicating Gag::eGFP presence. Dotted grey line: conductivity. (**C**): SDS-Page of samples collected after each purification step: harvest, primary clarification, secondary clarification, ion exchange E2 fraction and size-exclusion VV fraction. A total protein concentration increase can be observed after the IEX step, caused mostly by undesired protein contaminants. As can be observed, those proteins are successfully eliminated after the following SEC polishing step. (**D**): Western blot of samples after purification steps. Top: Membrane was treated with an anti-SARS-CoV-2 S2 spike subunit polyclonal antibody, followed by a goat anti-rabbit secondary incubation. Bottom: Membrane was treated with an anti-HIV-1 p24 monoclonal antibody, followed by a goat anti-mouse secondary incubation. Purified S-VLPs and G-VLPs show HIV-1 Gag bands, while only S-VLPs show SARS-CoV-2 S protein bands.

The capture step consisted of an anion exchange (IEX) chromatography to separate molecules based on their net surface charge, concentrating the desired S-VLPs while decreasing the contaminants’ contents. As the ion concentration was changed in different steps, the expected elution peaks were observed in the chromatograms at 488 nm absorbance (Figure 5A). Absorbance at 488 nm is caused by Gag::eGFP proteins, which allow monitoring S-VLP presence at the different stages of the process. The desired highly VLP-concentrated elution peak was collected and loaded into the next step.

The size-exclusion (SEC) polishing step is intended for bulk impurities removal, the elimination of VLP aggregates, desalting and buffer exchange to achieve the final product with the desired level of purity [31]. The polishing step was performed successfully, as the chromatograms show (Figure 5B). Two main 488nm peaks can be observed: the first one is the void volume fraction, which was collected as the final product. The second peak corresponds to contaminants “C” fraction and contains undesired VLP aggregates as analyzed by NTA (data not shown), which explains the notably high 488 nm absorbance levels.

As shown in Table 2, although a significant loss in terms of total number of VLPs, especially due to the low yield during the capture step, the overall DSP increased the VLP concentration from the initial bulk to the final product. The capture and polishing steps also had a significant positive impact in increasing the VLP purity over total particles, from 17.9% at the harvest to 31.1% at the final product.

The whole downstream process also succeeded in the reduction of undesired residual cellular contaminants, a crucial quality requirement for vaccines produced in cellular platforms [32]. The final purified sample contains 1.03% of the initial DNA and 0.22% of the initial protein (Table 2). This reduction of undesired protein concentration can also be observed by SDS-Page, especially after the polishing step (Figure 5C).

Along the different purification process steps, a drop in spike concentration higher than that expected due to VLP loss can be observed (Table 2), as analyzed by dot blot. This can be explained by the fact that, after VLP budding, unincorporated spike protein will remain present in the cellular membrane of the cells and be present in the cellular debris. This fraction is then removed during clarification steps. In addition, spike monomers solved at the clarified bulk are removed during the following capture and polishing steps. These decreasing values indicate that the S protein present at the final formulation could be due to properly VLP-incorporated and folded S proteins, which will present the immunogenic epitopes to the vaccinated patient’s immune system in a disposition that resembles the native S protein present at the SARS-CoV-2 virus. Western blot analyses for different DSP fractions using antibodies against HIV-1 Gag and SARS-CoV-2 spike confirmed the presence of those proteins with no significant migration pattern changes along the purification process (Figure 5D). The final purified product was also compared with identically purified G-VLPs, showing an almost identical Gag pattern together with no spike presence, as was expected for the negative control.

Although the overall purification process shows very good purification results in terms of purity, a further optimization of the capture step would help to establish a methodology that will increase downstream process yield by reducing VLP loss.

## 4. Discussion

In this article, we first evaluated the cellular co-expression of SARS-CoV-2 spike glycoprotein with HIV-1 Gag, concluding that it has no significant negative effect in cell growth and viability. This suggests that it has no cytotoxic effect caused by protein secretory pathway failures. Confocal microscopy analysis showed that, after its expression, native envelope spike glycoprotein travels to the plasmatic membrane, wherein it co-localizes with Gag::eGFP. As Gag-based VLP generation occurs at the plasmatic membrane via budding, those results lay the groundwork to hypothesize that the S protein is incorporated to the VLPs. After that, we analyzed the produced and sucrose-cushion-purified VLPs by Western blot to find that S protein is present on the produced Gag-VLPs, confirming the incorporation of this SARS-CoV-2 antigen in our vaccine candidate. The produced VLPs had a mode diameter of 134.9±1.2 nm, as measured by NTA. EM observations led us to conclude that they had no significant structural differences from Gag-based non-functionalized G-VLPs. Further, S protein presence was confirmed by immunogold labeling at the surface of S-VLPs, a key feature in order to present immunogenic SARS-CoV-2 epitopes to a patient’s immune system when used as a vaccine. This was also relevant as, to the best of our knowledge, this study is the first report of Gag-based VLP functionalization with SARS-CoV-2 epitopes in order to generate a vaccine candidate against COVID-19.

Further, we optimized the production bioprocess using design of experiments in order to increase S-VLP productivity. We identified the transfection conditions maximizing the cellular population co-expressing simultaneously Gag and S proteins. This is important in order to maximize the percentage of cells responsible for the production of the S-VLPs and to minimize the single-expressing population that generates non-functionalized VLPs. The model predicted a double-positive population of 57.5±2.3% for the optimal transfection condition, which was validated and then implemented to transfect a 1L stirred tank bioreactor.

The bioprocess was carried out satisfactorily, achieving good cellular growth and viabilities comparable to the parallel Erlenmeyer shake flasks. The double-transfected population accounted for 55.1% of the total cells, which is concordant with what was predicted by the previously generated model. The bioreactor showed similar behavior with the parallel Erlenmeyer flasks at 72 hpt in terms of spike concentration, VLP concentration and the purity of the produced S-VLPs. This confirmed that production in a 1 L reactor was achieved successfully.

Finally, the 1 L bioreactor harvested product was purified using a downstream process consisting of two clarification steps, an anion-exchange capture step and a size-exclusion final polishing step. Clarification steps succeeded at reducing the turbidity of the sample by removing undesired contaminants, aggregates, intact cells and debris. Capture and polishing steps reduced the presence of undesired proteins, dsDNA and VLP aggregates while increasing VLP purity. The final purified product presents a significant reduction of dsDNA (1.03%) and host cell protein presence (0.22%) relative from the initial sample. Western blot analysis helped to track spike and Gag presence along the purification process, while dot blot analyses were also performed in order to quantify spike concentrations, obtaining a concentration of 2.198 ng/µL at the final purified product. Overall, the DSP process had a low yield in terms of VLP recovery but highly succeeded at concentrating and purifying the desired S-VLPs while generating a final product with little undesired contaminant presence.

## 5. Conclusions

This work established a method for the production of SARS-CoV-2 VLPs by their co-expression with Gag::eGFP. We studied their expression, demonstrated the incorporation of the desired S proteins onto the produced VLPs and optimized the production process. Then, we successfully performed the bioprocess at a 1 L bioreactor scale and purified the produced harvest using a scalable DSP process. Furthermore, the reagents used in this work were animal-free, and all the materials and equipment used throughout the whole bioprocess ere cGMP. This facilitates the potential transfer of the product manufacture to the industrial scale.

The bioprocess defined in this work will be subsequently used to produce new VLP candidates against recently emerged COVID-19 variants, since it harbors the potential to produce different Gag-based chimeric VLPs. The future work will also be focused on testing the produced S-VLPs by evaluating its immunogenic potential against convalescent COVID-19 patient sera and mice animal model.

## Figures and Tables

**Table 1:** Box–Behnken experimental design: code levels, matrix design, response and regression coefficients.

**Independent Variables**			**Coding Levels**		
			**−1**	**0**	1
pGag::eGFP (µg/mL)			0.2	0.8	1.4
pSpike (µg/mL)			0.2	0.8	1.4
PEI (µg/mL)			2	3.5	5
**Number**		**pGag::eGFP (X_1_)**	**pSpike (X_2_)**	**PEI (X_3_)**	**Double-Positive Population ^a^ (%)**
1		0	0	0	46.9
2		1	0	1	43.1
3		0	0	0	47.2
4		−1	−1	0	36.5
5		0	−1	1	44.9
6		1	−1	0	28.3
7		−1	0	1	42.7
8		0	0	0	47.8
9		0	1	−1	51.3
10		−1	1	0	50.1
11		−1	0	−1	56.4
12		0	−1	−1	46.4
13		1	1	0	37.2
14		0	1	1	50.3
15		1	0	−1	39.0
		**Factor**	**Coefficient**	**f Value**	***p* Value**
		Intercept	47.3		
		A-pGag::eGFP	−4.7625	33.078	0.0022
		B-pSpike	4.1	24.516	0.0043
		C-PEI	−1.5125	3.336	0.1273
		AB	−1.175	1.007	0.3617
		AC	4.45	14.440	0.0126
		BC	0.125	0.011	0.9191
		A^2	−6.1	25.046	0.0041
		B^2	−3.175	6.785	0.0480
		C^2	4.1	11.315	0.0200
	**DF**	**SS**	**MS**	**F value**	***p*-value**
Model	9	668.29	74.25	13.536	0.0052
Error	2	0.42	0.21		

Abbreviations: DF degree of freedom, SS sum of squares, MS mean square. ^a^ Response is ICC-stained double-positive population at 72 h post transfection.

**Table 2:** Data of the different purification steps.

Step	Volume (mL)	Total Protein (mg)	Protein Yield (%) ^a^	Total DNA (µg)	DNA Yield (%) ^a^	VLP Conc. (VLPs/mL)	VLP Purity (%)	Total VLPs	VLP Yield (%) ^b^	Spike Conc. (µg/mL)
Harvest	1000	1074.9	100	2232.9	100	2.76 × 10^9^	17.9	2.76 × 10^12^	-	1.780
Clarified 1	965	958.5	89.17	1558.0	69.77	2.94 × 10^9^	21.2	2.84 × 10^12^	102.79	1.000
Clarified 2	960	930.5	86.57	1457.4	65.27	2.35 × 10^9^	17.9	2.26 × 10^12^	79.52	0.970
Ion Ex.	6	7.1	0.66	553.7	24.80	3.53 × 10^9^	24.9	2.12 × 10^11^	10.01	5.828
Size Ex.	7	0.2	0.02	23.1	1.03	8.61 × 10^9^	31.3	6.03 × 10^10^	37.94	2.198

^a^ From initial step. ^b^ From previous step.
